# Endovascular Embolization Through Grafts for Recurrent Giant Complex Internal Carotid Artery Aneurysms After Combined Microsurgical Low-Flow With Subsequent High-Flow Revascularization and Parent Artery Occlusion

**DOI:** 10.1227/neuprac.0000000000000119

**Published:** 2024-10-10

**Authors:** Chingiz Nurimanov, Karashash Menlibayeva, Iroda Mammadinova, Nurtay Nurakay, Yerbol Makhambetov

**Affiliations:** * Department of Vascular and Functional Neurosurgery, National Centre for Neurosurgery, Astana, Kazakhstan;; ‡ Department of Hospital Management, National Centre for Neurosurgery, Astana, Kazakhstan

**Keywords:** Complex giant aneurysms, Cerebral revascularization, Recanalization, Parent artery occlusion, Hybrid approach, Endovascular embolization

## Abstract

**BACKGROUND AND IMPORTANCE:**

The case describes the management of a giant and recurrent aneurysm in a patient. The treatment involved a combination of microsurgical protective low-flow, followed by high-flow revascularization and parent artery occlusion.

**CLINICAL PRESENTATION:**

The patient presented with severe headaches and progressive visual impairment. Brain magnetic resonance imaging revealed a giant saccular intracranial aneurysm in the left internal carotid artery. The examination also revealed hypoplasia of the right anterior cerebral artery and a fetal-type left posterior cerebral artery originating from the neck of the aneurysm. Considering the location and size of the aneurysm, a combination of endovascular and microsurgical approaches was applied. A protective low-flow procedure was followed by high-flow revascularization and occlusion of the parent artery. After 12 months, the aneurysm recanalized through the graft. To address the recurrence of giant complex aneurysms, endovascular embolization was performed through the radial artery grafts. These interventions showed positive midterm outcomes for the patient's giant aneurysm.

**CONCLUSION:**

In the treatment of complex giant aneurysms, it is important to consider both endovascular and microsurgical approaches as complementary, rather than mutually exclusive. Endovascular embolization using radial artery grafts may be used when dealing with retrograde recanalization and the challenges associated with accessing the aneurysm.

**ABBREVIATIONS:**
PCA: posterior cerebral artery
RA: radial artery
STA: superior temporal artery.

Managing complex cerebral aneurysms remains a persistent challenge despite advancements in microsurgical and endovascular techniques.^1-3^ The treatment options for these aneurysms involve a variety of endovascular and microsurgical methods, which are often associated with increased rates of mortality and morbidity.^4^

Difficulties commonly arise from the frequent occurrence of intraoperative complications and procedural challenges.^5^ Therapy involving a multidisciplinary team of endovascular and neurovascular surgeons or specialists who are trained in both disciplines has shown promising results, with low rates of intrasurgery and postsurgery complications.^6^ Specifically, high-flow or low-flow bypass combined with subsequent endovascular parent artery occlusion using flow reversal has demonstrated positive postsurgery outcomes.^2,7^

Nevertheless, there is lack of data on recurrence rates after the use of combined approaches and treatment methods. This case presents the treatment strategy and the results authors have achieved in managing the complex and rare case.

## CLINICAL PRESENTATION

**FIGURE 1.**
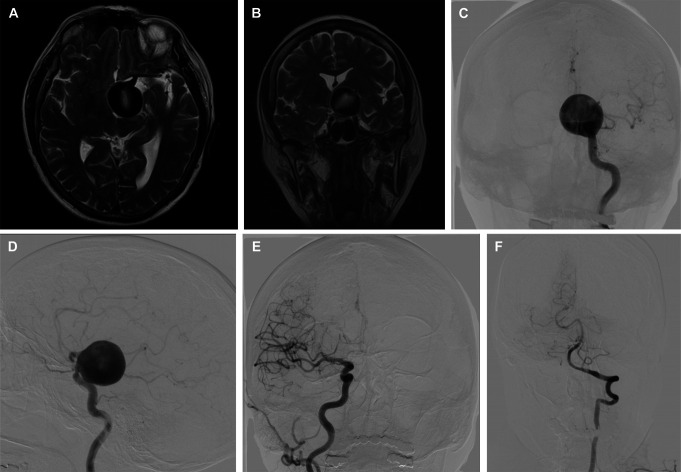
Brain MRIs. **A**, Axial and **B**, frontal T2-weighted images demonstrating the giant intracranial aneurysm of the left ICA. **C** and **D**, Carotid angiograms from the left ICA show a giant saccular aneurysm in the paraclinoid segment of the left ICA. **E**, Carotid angiogram from the right ICA reveals hypoplasia of the right anterior cerebral artery **F**, An angiogram from the left vertebral artery shows a fetal type of left posterior cerebral artery. ICA, internal carotid artery.

A 66-year-old right-handed woman was admitted to the hospital due to headaches and progressive visual impairment in the left eye over the past 6 months. An examination revealed no neurological deficits at the hospital. A brain MRI displayed a giant saccular intracranial aneurysm in the left internal carotid artery (ICA) measuring 31.22 × 28.68 × 27.52 mm (Figure 1A and 1B), which was further confirmed through digital subtraction angiography (DSA) (Figure 1C and 1D) and hypoplasia of the right anterior cerebral artery (ACA) (Figure 1E), a fetal-type left posterior cerebral artery (PCA) (Figure 1F) originating from the neck of the aneurysm.

Endovascular embolization and microsurgical clipping were considered not appropriate. Therefore, a combined protective low-flow with subsequent high-flow extra-intracranial bypass was scheduled to restore the blood supply to the left ACA, middle cerebral artery (MCA), and fetal-type PCA, followed by parent artery occlusion using an endovascular approach.

**FIGURE 2.**
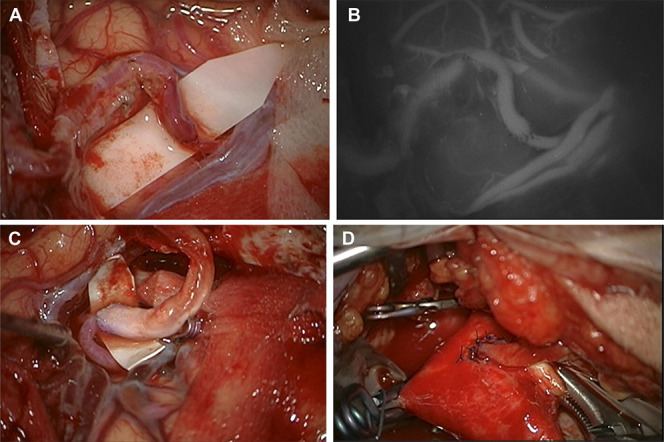
Intraoperative view. **A**, Low-flow “end-to-side” superior temporal artery–M4 segment MCA bypass. **B**, Indocyanine Green angiography showing patency of low-flow bypass. **C**, “End-to-side” distal RA graft—M2 segment MCA bypass. **D**, “End-to-side” proximal RA graft—external carotid artery bypass. MCA, middle cerebral artery; RA, radial artery.

The first procedure was the protective low-flow superior temporal artery (STA)–MCA bypass, which involved connecting the frontal branch of the left STA to the M4 segment of the left ICA (Figure 2A and 2B). After that, the radial artery (RA) graft was prepared from the left arm. The RA graft was tunneled under the zygomatic arch to the left temporal region. End-to-side anastomoses were performed between the cranial part of the graft and the M2 segment of the left MCA (Figure 2C) and between the cervical part of the graft and the external carotid artery above the common carotid artery bifurcation (Figure 2D). Intraoperative indocyanine green video angiography was used to confirm the bypass patency.

**FIGURE 3.**
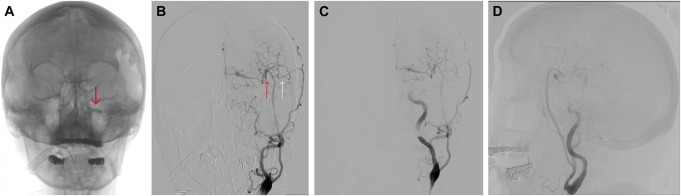
Digital subtraction angiography. **A**, A carotid angiogram from the left CCA shows balloon occlusion of the left ICA (red arrow points to the balloon in ICA). **B**, Patency of the low-flow (white arrow) and high-flow (red arrow) bypasses. **C** and **D**, a carotid angiogram from left CCA after parent artery (ICA) coil occlusion. CCA, common carotid artery; ICA, internal carotid artery.

**FIGURE 4.**
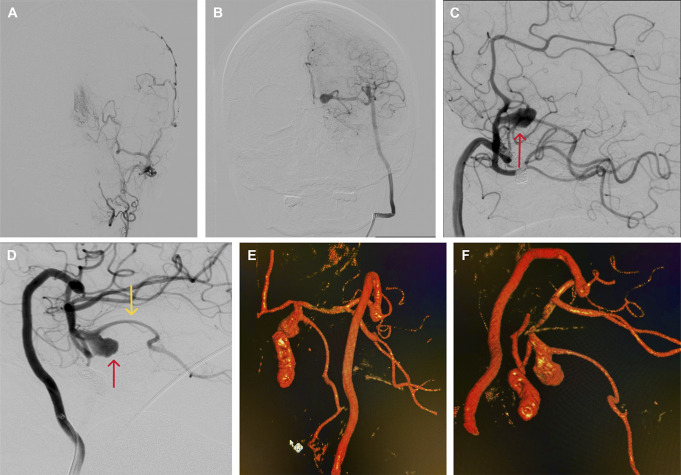
Digital subtraction angiography. **A**, A carotid angiogram from the left external carotid artery revealed low-flow bypass patency. **B**, An angiogram from the RA graft, shows the patency of the bypasses, with the left carotid territory supplied through high-flow bypasses. **C** and **D**, an angiogram from the RA graft revealing aneurysm neck recanalization (red arrow) and fetal type posterior cerebral artery (yellow arrow) originating from the aneurysm neck (LAO 90, CAU 36 and LAO 93, CAU 34). **E** and **F**, Three-dimensional reconstructions confirm the aneurysm recanalization. CAU, cauda; LAO, left anterior oblique; RA, radial artery.

After the bypass, the balloon occlusion test confirmed good graft patency (Figure 3A and 3B). After that, endovascular coil occlusion was performed on the ophthalmic segment of the left ICA to prevent retrograde filling. Postoperative cerebral angiography demonstrated total occlusion of the cavernous and ophthalmic segments of the left ICA, with supply to the left ACA, MCA, and PCA through the bypasses (Figure 3C and 3D), and a good patency of protective STA-MCA (Figure 4A). She was discharged in good condition and had a modified Rankin Scale score of 0.

**FIGURE 5.**
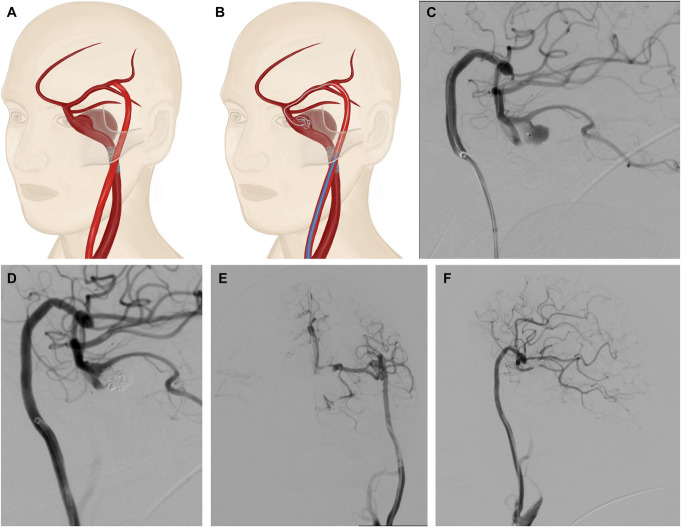
**A** and **B**, Illustration of the aneurysm recanalization and embolization through the radial graft. **C**, Angiogram from the RA graft (LAO 93, CAU 34) demonstrating aneurysm microcatheterization. **D**, Angiogram from the RA graft (LAO 92, CAU 28) illustrating aneurysm coiling. **E** and **F**, Control angiography from the RA graft showing total occlusion of the aneurysm and distal internal carotid artery. CAU, cauda; LAO, left anterior oblique; RA, radial artery.

After 12 months, a DSA was performed, which revealed a “stop-contrast” at the cervical segment of the left ICA, with perfusion of the left ACA, MCA, and fetal PCA through the bypasses. The high-flow bypass detected retrograde recanalization of the aneurysm neck, which measured 14.95 × 8.08 × 5.38 mm (Figure 4B-4F, 5A).

It was decided to embolize the recanalized aneurysm neck and distal ICA through the RA graft (Figure 5B-5D). For this, a 5-Fr guide catheter was placed in the extracranial segment of the graft, and 3000 units of heparin were administered intravenously. A microcatheter was then advanced through the high-flow anastomosis to the residual aneurysm, using a micro-guidewire. The embolization procedure was performed using detachable coils (Figure 5C and 5D).

Postoperative DSA confirmed the patency of the bypass and the absence of any residual aneurysm (Raymond-Roy I) (Figure 5E and 5F). The patient was discharged with a modified Rankin Scale score of 0 on the next day.

### Ethics Committee Statement

The Institutional Review Board of the National Centre for Neurosurgery has approved the study, and the patient has given written informed consent for the publication of this case.

## DISCUSSION

In this study, the outcomes of surgical interventions for recanalized complex ICA aneurysms in a patient after a combined endovascular and open microsurgery approach are presented.

Giant complex cerebral aneurysms present significant challenges in neurovascular care.^8,9^ Because of their natural progression and the potential for severe clinical consequences, it is agreed upon that timely and comprehensive treatment is crucial.^10^ However, treatment strategies need to be customized to each patient's specific condition because there is no universal approach that guarantees success.^11^

At our institution, we perform STA-MCA protective and high-flow bypasses using radial grafts. After this, we use endovascular parent artery occlusion in the proximal ICA for patients with complex aneurysms, particularly when it is necessary to supply 3 main cerebral artery territories.^12^ Although there is extensive documentation on endovascular embolization after microsurgical clipping,^13^ this particular case is unique because it involves retrograde recanalization and presents additional challenges in approaching the aneurysm. Previously, Chwajol et al^14^ described 3 cases of embolization of posterior circulation aneurysms through carotid-vertebral artery bypass. However, there are no reported cases in the literature of embolization of a recanalized internal ICA aneurysm through a high-flow bypass RA graft. Scholars argue that postoperative recanalization and rupture can occur because of the retrograde filling of the aneurysm by the bypass graft or by ICA branches, such as the ophthalmic artery or the meningohypophyseal trunk.^15,16^ To prevent these complications, a dense coiling of aneurysms is recommended.^17,18^ However, in the case of giant aneurysms located near cranial nerves, this approach is not safe because of the mass effect. In our approach, we avoided the mass effect by tightly packing coils in the proximal ICA and confirmed the occlusion of the aneurysm through angiography.

A high-flow anastomosis can potentially increase the risk of retrograde aneurysm recanalization. This is due to the elevated hemodynamic stress, especially near a fetal PCA. According to Wiśniewski et al,^19^ the morphometric parameters of the aneurysm contribute to recanalization by creating a complex flow pattern in the aneurysmal sac and any adjacent branches.

Surgical intervention is strongly recommended for patients diagnosed with recanalized and growing aneurysms because they are at an increased risk of rupture.^20^ However, traditional surgical techniques such as direct clipping carry inherent risks in cases of complexly positioned aneurysms or those previously treated with bypass procedures. Open surgery can disrupt compensatory vascular bypasses, causing subsequent complications. As a result, endovascular treatment becomes the primary method for addressing these types of aneurysms.^21,22^ A recent systematic review evaluating secondary coiling after incomplete microsurgery of cerebral aneurysms showed positive outcomes, underscoring the preference for endovascular strategies in complex cases.^13^

Significant difficulties arose in accessing the aneurysm due to the occlusion of the ICA by endovascular approach. Although accessing the contralateral aneurysm through the communicating arteries is feasible,^23^ this approach becomes unattainable when hypoplasia or aplasia of these arteries is present, as was in a reported case.

The introduction of flow diverters has improved the treatment of giant aneurysms. However, in the case of serpentine or fusiform aneurysms, the risk of recurrence is significant, and current interventions have not proven to be effective.^24^ Furthermore, the discrepancy in diameters between the ICA and the PCA, along with the need for larger diameter microcatheters for stent deployment compared with coiling, present additional challenges.

## CONCLUSION

Endovascular embolization using RA grafts may be used in cases where retrograde recanalization and difficulties in reaching the aneurysm are present. When treating complex giant aneurysms, it is important to consider both endovascular and microsurgical approaches as complementary rather than mutually exclusive.

## COMMENTS

This case report provides an instructive example of the complex multistage management required for giant intracranial aneurysms. The authors detail their approach combining microsurgical revascularization with endovascular techniques to treat a challenging left internal carotid artery aneurysm.

The initial low-flow and high-flow bypass strategy followed by parent artery occlusion demonstrates sound neurovascular principles. The authors appropriately considered the patient's anatomy in their treatment planning, including the fetal posterior cerebral artery arising from the aneurysm neck. The subsequent retrograde recanalization highlights an essential consideration with high-flow bypass procedures.

An essential contribution of this report is the groundbreaking use of endovascular embolization through the radial artery graft to treat the recanalized aneurysm. As the authors note, this technique, the first for an internal carotid artery aneurysm, significantly expands the treatment options for complex aneurysms with limited access.

The case underscores several points that could be considered: consideration of distal aneurysm clipping during the initial bypass may have prevented recanalization. Flow diversion could have been considered an initial treatment, though the authors provide a reasonable rationale for their approach given the patient's anatomy. The successful outcome supports the reviewers' comments and validates the value of multimodality management for these challenging lesions. Some limitations are worth noting. As a single case report, the generalizability of this approach remains to be determined. In addition, longer-term follow-up would be valuable to assess the durability of the intervention.

Overall, this report contributes to the literature on complex aneurysm management. It reinforces the importance of tailoring treatment strategies to individual patient factors. It demonstrates how the creative application of endovascular and open techniques can address challenges during treatment. The case should encourage neurovascular specialists to consider all available options when faced with the recanalization of previously treated complex aneurysms.

**Clemens M. Schirmer**

*Wilkes-Barre, Pennsylvania, USA*
